# Supplementary material for: Contrasting nidification behaviors facilitate diversification and colonization of the Music frogs under a changing paleoclimate
Source: Commun Biol. 2024 May 25;7:638. doi: 10.1038/s42003-024-06347-7 (PMC11127999; doi:10.1038/s42003-024-06347-7)
Supplement: Supplementary file 2 — Supplementary Information [file 42003_2024_6347_MOESM2_ESM.pdf]

Supplemental Information for:

Contrasting nidification behaviors facilitate diversification and colonization of the Music frogs under a changing paleoclimate

Zhi-Tong Lyu, Zhao-Chi Zeng, Han Wan, Qin Li, Atsushi Tominaga, Kanto Nishikawa, Masafumi Matsui, Shi-Ze Li, Zhong-Wen Jiang, Yang Liu, Ying-Yong Wang

Contents

**Supplementary Figures**	Page 2
**Supplementary Tables**	
Supplementary Table S1	Page 6
Supplementary Table S2	Page 16
Supplementary Table S3	Page 17
Supplementary Table S4	Page 18

Supplementary Figures

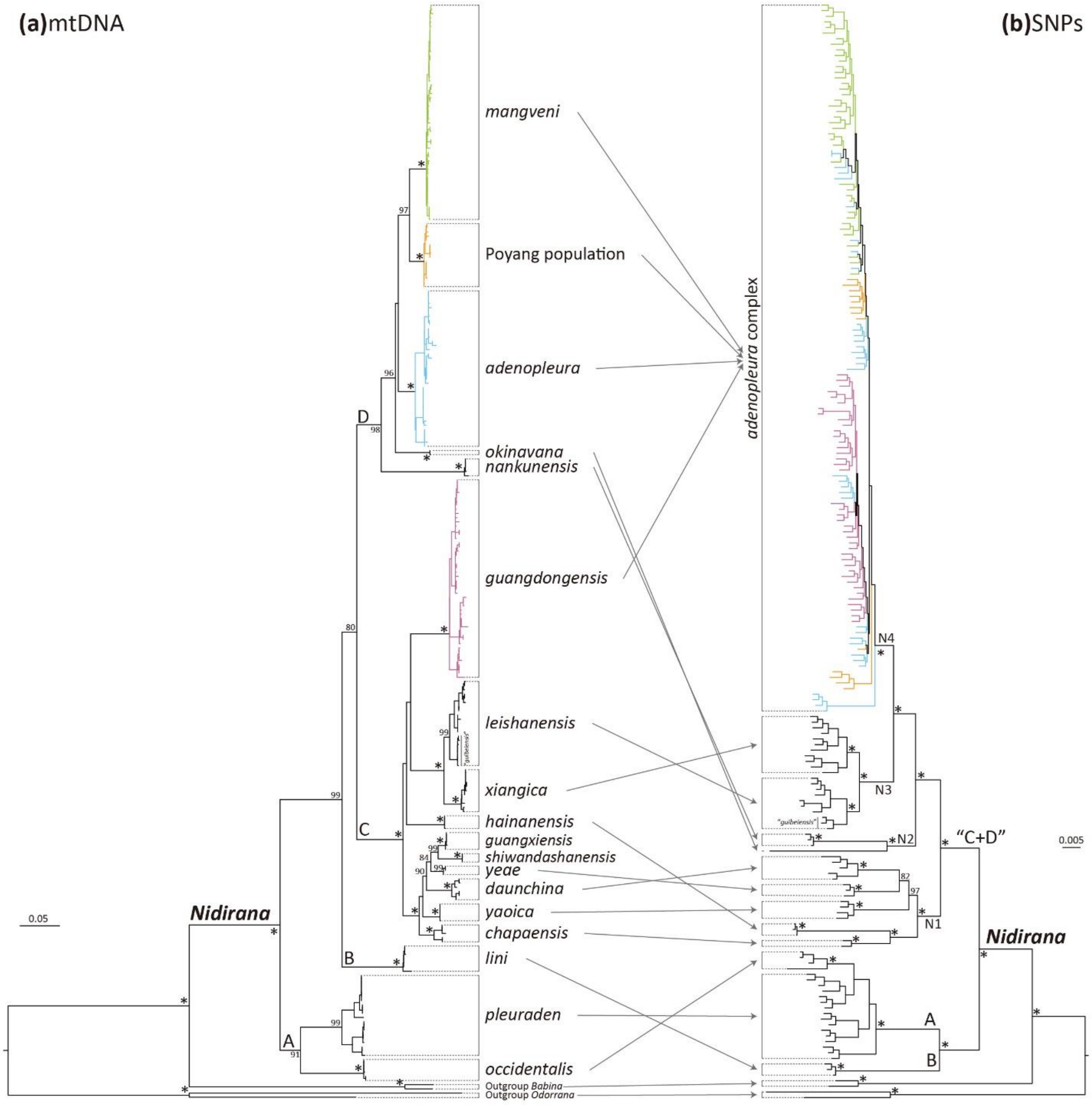

Supplementary Figure S1 Phylogeny of *Nidirana*, using maximum likelihood based on mitochondrial genes of 16S and COI **(a)** and based on SNPs data **(b)**, respectively. Bootstrap supports (BS) larger than 80 are shown, and asterisks represent BS = 100.

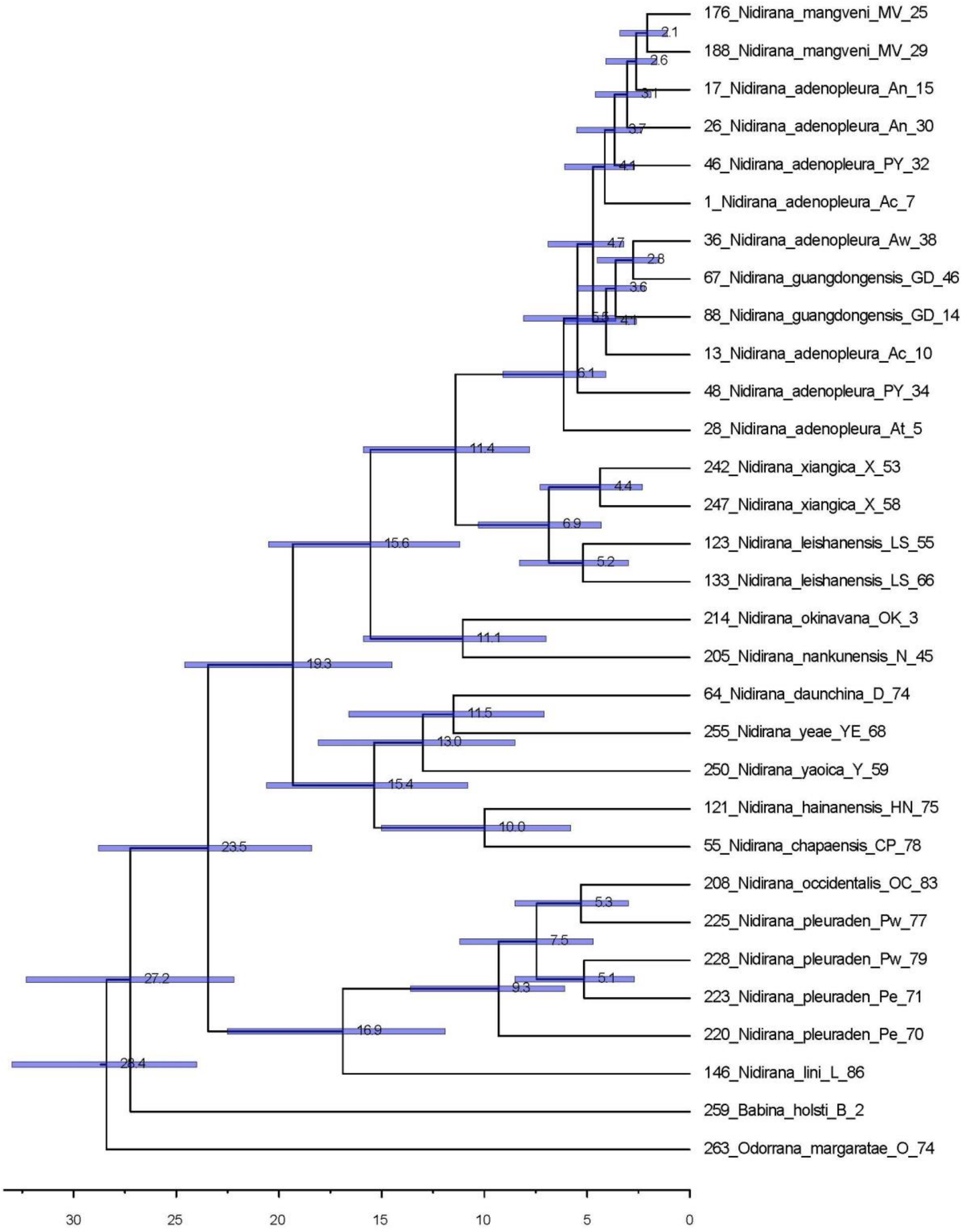

Supplementary Figure S2 Estimated divergence time for the genus *Nidirana*. Samples are labeled as “sample ID_species name_Population_Locaility ID”, which are corresponding to the information in Supplementary Table S1.

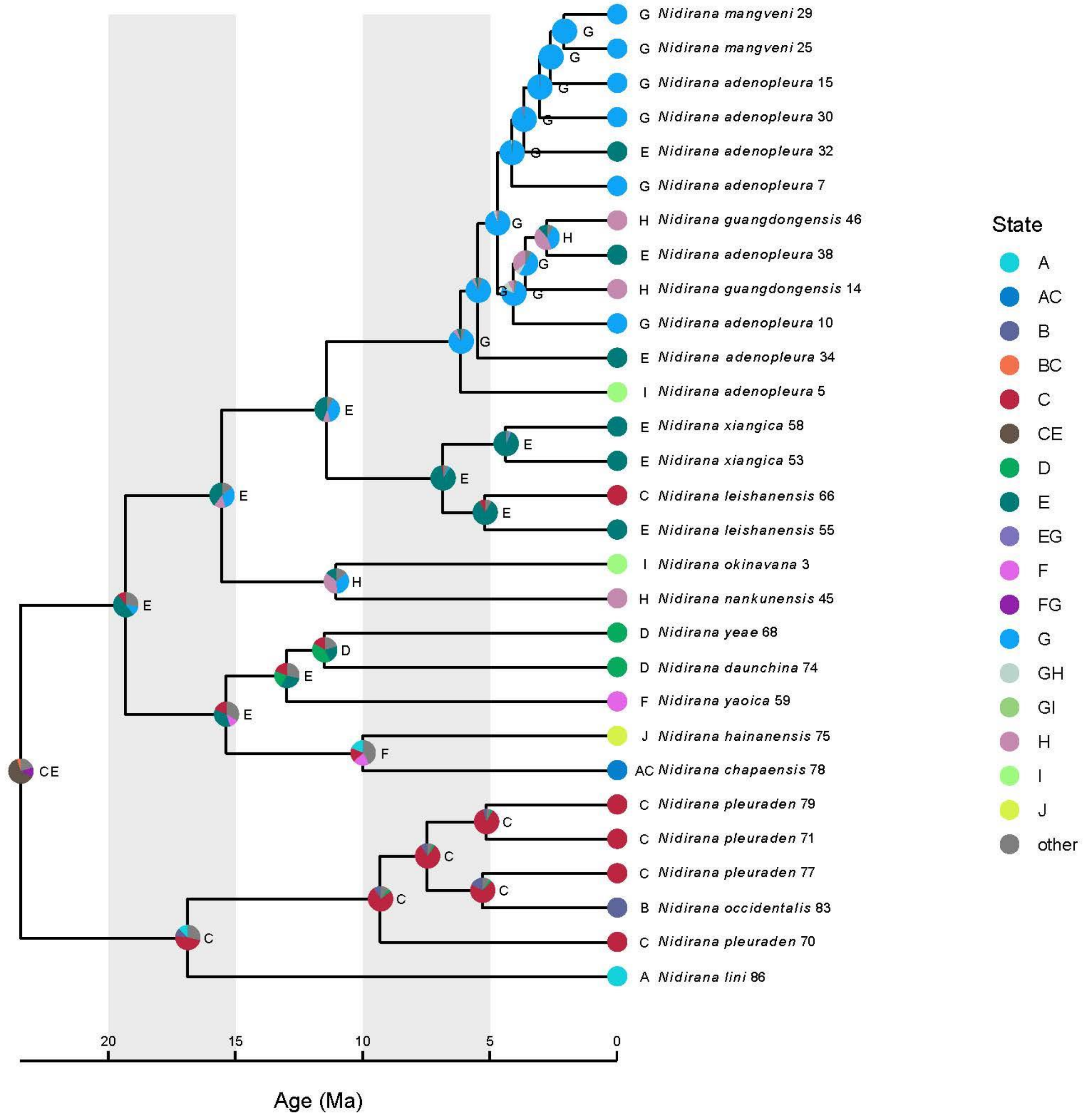

Supplementary Figure S3 Ancestral distributions for the genus *Nidirana* with pie charts for the proportional likelihoods of ancestral area states. Samples are labeled as "species name_Locaility ID", which are corresponding to the information in Supplementary Table S1. Characters for geographical areas are defined in Supplementary Table S2.

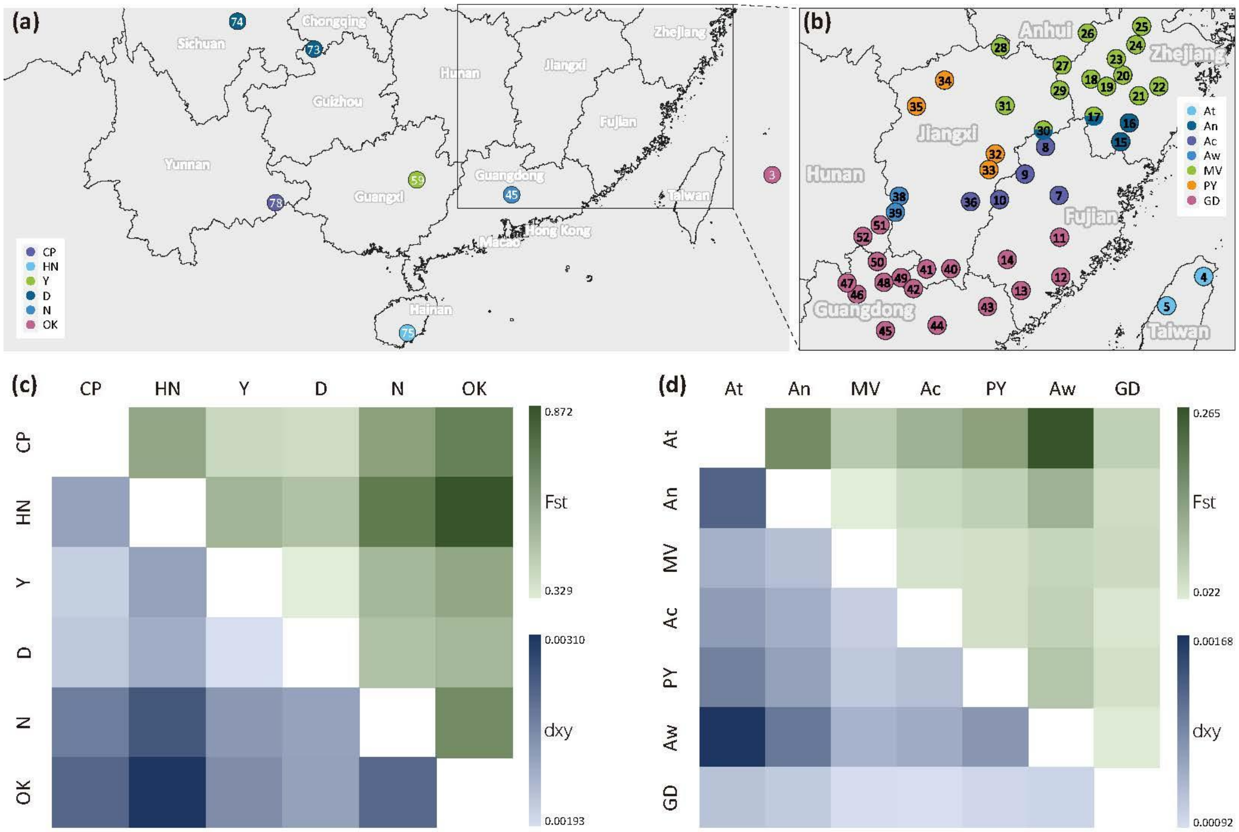

Supplementary Figure S4 **(a)** Map showing collected locality for the nidification species. **(b)** Map showing collected locality for the *N. adenopleura* complex. Numbers for localities are corresponding to the information in Supplementary Table S1. **(c)** Wright's fixation index (Fst) and absolute genetic divergences (dxy) statistics among the six nidification species. **(d)** Wright's fixation index (Fst) and absolute genetic divergences (dxy) statistics among the seven population of the *N. adenopleura* complex. Labels for different populations: *N. chapaensis* (CP), *N. hainanensis* (HN), *N. yaoica* (Y), *N. daunchina* (D), *N. nankunensis* (N), *N. okinavana* (OK), *N. mangveni* (MV), the Poyang population (PY), *N. guangdongensis* (GD), and four populations of *N. adenopleura*: Taiwan (At), north (An), center (Ac), west (Aw).

Supplementary Tables

Supplementary Table S1 Localities, vouchers, and GenBank numbers for all samples used in this study. Population labels are majorly abbreviated from species nomenclatures, except for: At – Taiwan population of *Nidirana adenopleura*, Ac – central population of *N. adenopleura*, An – northern population of *N. adenopleura*, Aw – western population of *N. adenopleura*, Au – samples of *N. adenopleura* that could not be assigned to a population due to deficient data, Pe – eastern population of *N. pleuraden*, and Aw – western population of *N. pleuraden*.

ID	Species	Population	Locality (Locality ID)	Voucher	16s rRNA	CO1	RAD-seq
1	*Nidirana adenopleura*	Ac	China: Fujian: Yanping (7)	SYS a005911	MF807844	MF807883	√
2	*Nidirana adenopleura*	Ac	China: Fujian: Yanping (7)	SYS a005913	MF807846	MF807885	√
3	*Nidirana adenopleura*	Ac	China: Fujian: Yanping (7)	SYS a005914	MF807847	MF807886	√
4	*Nidirana adenopleura*	Ac	China: Fujian: Yanping (7)	SYS a005915	MF807848	MF807887	√
5	*Nidirana adenopleura*	Ac	China: Fujian: Yanping (7)	SYS a005916	MF807849	MF807888	√
6	*Nidirana adenopleura*	Ac	China: Fujian: Mt Wuyi (8)	SYS a005939	MF807850	MF807889	√
7	*Nidirana adenopleura*	Ac	China: Fujian: Mt Wuyi (8)	SYS a005940	MF807851	MF807890	√
8	*Nidirana adenopleura*	Ac	China: Fujian: Mt Wuyi (8)	SYS a005941	MF807852	MF807891	√
9	*Nidirana adenopleura*	Ac	China: Fujian: Shaowu (9)	SYS a004112	MF807833	MF807872	√
10	*Nidirana adenopleura*	Ac	China: Fujian: Shaowu (9)	SYS a004132	MF807834	MF807873	√
11	*Nidirana adenopleura*	Ac	China: Fujian: Mt Yashu (10)	SYS a005891	MF807841	MF807880	√
12	*Nidirana adenopleura*	Ac	China: Fujian: Mt Yashu (10)	SYS a005901	MF807842	MF807881	√
13	*Nidirana adenopleura*	Ac	China: Fujian: Mt Yashu (10)	SYS a005902	MF807843	MF807882	√
14	*Nidirana adenopleura*	Ac	China: Jiangxi: Ningdu (36)	SYS a007089	MN946448	MN945204	√
15	*Nidirana adenopleura*	Ac	China: Jiangxi: Ningdu (36)	SYS a007090	MN946449	MN945205	√
16	*Nidirana adenopleura*	Ac	China: Jiangxi: Ningdu (36)	SYS a007091	MN946450	MN945206	√
17	*Nidirana adenopleura*	An	China: Zhejiang: Jingning (15)	SYS a002725	MF807827	MF807866	√
18	*Nidirana adenopleura*	An	China: Zhejiang: Liandu (16)	SYS a008450	ON391793	ON381784	
19	*Nidirana adenopleura*	An	China: Zhejiang: Suichang (17)	SYS a008039	ON391785	ON381776	√
20	*Nidirana adenopleura*	An	China: Zhejiang: Suichang (17)	SYS a008041	ON391786	ON381777	√
21	*Nidirana adenopleura*	An	China: Zhejiang: Suichang (17)	SYS a008042	ON391787	ON381778	√
22	*Nidirana adenopleura*	An	China: Zhejiang: Suichang (17)	SYS a008049	ON391788	ON381779	√
23	*Nidirana adenopleura*	An	China: Jiangxi: Yanshan (30)	SYS a008088	ON391789	ON381780	√

ID	Species	Population	Locality (Locality ID)	Voucher	16s rRNA	CO1	RAD-seq
24	*Nidirana adenopleura*	An	China: Jiangxi: Yanshan (30)	SYS a008101	ON391790	ON381781	√
25	*Nidirana adenopleura*	An	China: Jiangxi: Yanshan (30)	SYS a008103	ON391791	ON381782	√
26	*Nidirana adenopleura*	An	China: Jiangxi: Yanshan (30)	SYS a008106	ON391792	ON381783	√
27	*Nidirana adenopleura*	At	China: Taiwan: New Taipei (4)	UMMZ 189963	DQ283117	/	
28	*Nidirana adenopleura*	At	China: Taiwan: Taichung (5)	SYS a007362	ON391781	ON381772	√
29	*Nidirana adenopleura*	At	China: Taiwan: Taichung (5)	SYS a007363	ON391782	ON381773	√
30	*Nidirana adenopleura*	At	China: Taiwan: Taichung (5)	SYS a007364	ON391783	ON381774	√
31	*Nidirana adenopleura*	At	China: Taiwan: Taichung (5)	SYS a007365	ON391784	ON381775	√
32	*Nidirana adenopleura*	Au	China: Fujian: Zherong (6)	SYS a008481	ON391794	ON381785	
33	*Nidirana adenopleura*	Aw	China: Jiangxi: Suichuan (39)	SYS a007094	ON391780	ON381771	√
34	*Nidirana adenopleura*	Aw	China: Jiangxi: Suichuan (39)	SYS a004450	MN946456	MN945212	√
35	*Nidirana adenopleura*	Aw	China: Jiangxi: Suichuan (39)	SYS a004451	MN946457	MN945213	
36	*Nidirana adenopleura*	Aw	China: Jiangxi: Mt Jinggang (38)	SYS a004025	MF807830	MF807869	√
37	*Nidirana adenopleura*	Aw	China: Jiangxi: Mt Jinggang (38)	SYS a004026	MF807831	MF807870	√
38	*Nidirana adenopleura*	Aw	China: Jiangxi: Mt Jinggang (38)	SYS a004027	MF807832	MF807871	√
39	*Nidirana adenopleura*	PY	China: Jiangxi: Nancheng (32)	SYS a007033	ON391874	ON381865	√
40	*Nidirana adenopleura*	PY	China: Jiangxi: Nancheng (32)	SYS a007035	ON391875	ON381866	√
41	*Nidirana adenopleura*	PY	China: Jiangxi: Nancheng (32)	SYS a007036	ON391876	ON381867	√
42	*Nidirana adenopleura*	PY	China: Jiangxi: Nancheng (32)	SYS a007040	ON391877	ON381868	√
43	*Nidirana adenopleura*	PY	China: Jiangxi: Nancheng (32)	SYS a007042	ON391878	ON381869	√
44	*Nidirana adenopleura*	PY	China: Jiangxi: Nancheng (32)	SYS a007043	ON391879	ON381870	√
45	*Nidirana adenopleura*	PY	China: Jiangxi: Nancheng (32)	SYS a007053	ON391880	ON381871	√
46	*Nidirana adenopleura*	PY	China: Jiangxi: Nancheng (32)	SYS a007055	ON391881	ON381872	√
47	*Nidirana adenopleura*	PY	China: Jiangxi: Nanfeng (33)	SYS a007073	ON391882	ON381873	√
48	*Nidirana adenopleura*	PY	China: Jiangxi: Mt Jiuling (34)	SYS a006974	ON391867	ON381858	√
49	*Nidirana adenopleura*	PY	China: Jiangxi: Mt Jiuling (34)	SYS a006975	ON391868	ON381859	√
50	*Nidirana adenopleura*	PY	China: Jiangxi: Mt Jiuling (34)	SYS a006976	ON391869	ON381860	√
51	*Nidirana adenopleura*	PY	China: Jiangxi: Mt Jiuling (34)	SYS a006977	ON391870	ON381861	√
52	*Nidirana adenopleura*	PY	China: Jiangxi: Yifeng (35)	SYS a007001	ON391871	ON381862	

ID	Species	Population	Locality (Locality ID)	Voucher	16s rRNA	CO1	RAD-seq
53	*Nidirana adenopleura*	PY	China: Jiangxi: Yifeng (35)	SYS a007002	ON391872	ON381863	
54	*Nidirana adenopleura*	PY	China: Jiangxi: Yifeng (35)	SYS a007004	ON391873	ON381864	
55	*Nidirana chapaensis*	CP	China: Yunnan: Xichou (78)	SYS a008303	ON391883	ON619576	√
56	*Nidirana chapaensis*	CP	China: Yunnan: Xichou (78)	SYS a008304	ON391884	ON619577	√
57	*Nidirana chapaensis*	CP	Vietnam: Lao Cai: Sapa (87)	MNHN 1999.5871	KR827710	/	
58	*Nidirana chapaensis*	CP	Vietnam: Lao Cai: Sapa (87)	MNHN 2000.485	KR827711	KR087625	
59	*Nidirana chapaensis*	CP	Vietnam: Lao Cai: Sapa (87)	ROM 28070	AF206460	/	
60	*Nidirana daunchina*	D	China: Chongqing: Liangping (72)	SYS a008564	ON391885	ON381874	
61	*Nidirana daunchina*	D	China: Sichuan: Hejiang (73)	SYS a004930	MF807824	MF807863	√
62	*Nidirana daunchina*	D	China: Sichuan: Hejiang (73)	SYS a004931	MF807825	MF807864	√
63	*Nidirana daunchina*	D	China: Sichuan: Hejiang (73)	SYS a004932	MF807826	MF807865	√
64	*Nidirana daunchina*	D	China: Sichuan: Mt Emei (74)	SYS a004594	MF807822	MF807861	√
65	*Nidirana daunchina*	D	China: Sichuan: Mt Emei (74)	SYS a004595	MF807823	MF807862	√
66	*Nidirana guangdongensis*	GD	China: Guangdong: Yingde (46)	SYS a005995	MN946408	MN945164	√
67	*Nidirana guangdongensis*	GD	China: Guangdong: Yingde (46)	SYS a005997	MN946410	MN945166	√
68	*Nidirana guangdongensis*	GD	China: Guangdong: Yingde (46)	SYS a005998	MN946411	MN945167	√
69	*Nidirana guangdongensis*	GD	China: Guangdong: Yingde (46)	SYS a006879	ON391922	ON381911	√
70	*Nidirana guangdongensis*	GD	China: Guangdong: Mt Tianjing (47)	SYS a006933	ON391923	ON381912	√
71	*Nidirana guangdongensis*	GD	China: Guangdong: Mt Tianjing (47)	SYS a006934	MN946414	MN945170	√
72	*Nidirana guangdongensis*	GD	China: Guangdong: Mt Tianjing (47)	SYS a006936	ON391924	ON381913	√
73	*Nidirana guangdongensis*	GD	China: Guangdong: Mt Longtou (48)	SYS a002772	ON391893	ON381882	√
74	*Nidirana guangdongensis*	GD	China: Guangdong: Mt Longtou (48)	SYS a002795	ON391894	ON381883	√
75	*Nidirana guangdongensis*	GD	China: Guangdong: Mt Chebaling (49)	SYS a007900	MN946416	MN945172	
76	*Nidirana guangdongensis*	GD	China: Guangdong: Mt Chebaling (49)	SYS a007901	MN946417	MN945173	
77	*Nidirana guangdongensis*	GD	China: Guangdong: Renhua (50)	SYS a008135	MN946418	MN945174	
78	*Nidirana guangdongensis*	GD	China: Guangdong: Renhua (50)	SYS a008136	MN946419	MN945175	
79	*Nidirana guangdongensis*	GD	China: Guangdong: Mt Nankun (45)	SYS a005720	MN946412	MN945168	
80	*Nidirana guangdongensis*	GD	China: Guangdong: Mt Nankun (45)	SYS a005721	MN946413	MN945169	√
81	*Nidirana guangdongensis*	GD	China: Guangdong: Mt Nankun (45)	SYS a005789	ON391905	ON381894	√

ID	Species	Population	Locality (Locality ID)	Voucher	16s rRNA	CO1	RAD-seq
82	*Nidirana guangdongensis*	GD	China: Guangdong: Dongyuan (44)	SYS a005950	ON391909	ON381898	√
83	*Nidirana guangdongensis*	GD	China: Guangdong: Dongyuan (44)	SYS a005951	ON391910	ON381899	√
84	*Nidirana guangdongensis*	GD	China: Guangdong: Dongyuan (44)	SYS a005952	ON391911	ON381900	√
85	*Nidirana guangdongensis*	GD	China: Guangdong: Mt Tongguzhang (43)	SYS a005809	ON391906	ON381895	√
86	*Nidirana guangdongensis*	GD	China: Guangdong: Mt Tongguzhang (43)	SYS a005810	ON391907	ON381896	√
87	*Nidirana guangdongensis*	GD	China: Guangdong: Mt Tongguzhang (43)	SYS a005811	ON391908	ON381897	√
88	*Nidirana guangdongensis*	GD	China: Fujian: Shanghang (14)	SYS a004102	ON391900	ON381889	√
89	*Nidirana guangdongensis*	GD	China: Fujian: Shanghang (14)	SYS a004103	ON391901	ON381890	√
90	*Nidirana guangdongensis*	GD	China: Fujian: Nanjing (13)	SYS a006026	ON391915	ON381904	√
91	*Nidirana guangdongensis*	GD	China: Fujian: Nanjing (13)	SYS a006027	ON391916	ON381905	√
92	*Nidirana guangdongensis*	GD	China: Fujian: Nanjing (13)	SYS a006028	ON391917	ON381906	√
93	*Nidirana guangdongensis*	GD	China: Fujian: Xiamen (12)	SYS a007906	ON391925	ON381914	√
94	*Nidirana guangdongensis*	GD	China: Fujian: Xiamen (12)	SYS a007907	ON391926	ON381915	√
95	*Nidirana guangdongensis*	GD	China: Fujian: Mt Daiyun (11)	SYS a006013	ON391912	ON381901	√
96	*Nidirana guangdongensis*	GD	China: Fujian: Mt Daiyun (11)	SYS a006015	ON391913	ON381902	√
97	*Nidirana guangdongensis*	GD	China: Fujian: Mt Daiyun (11)	SYS a006016	ON391914	ON381903	√
98	*Nidirana guangdongensis*	GD	China: Jiangxi: Mt Sanbai (40)	SYS a003735	ON391895	ON381884	√
99	*Nidirana guangdongensis*	GD	China: Jiangxi: Mt Sanbai (40)	SYS a003736	ON391896	ON381885	√
100	*Nidirana guangdongensis*	GD	China: Jiangxi: Mt Sanbai (40)	SYS a003737	ON391897	ON381886	√
101	*Nidirana guangdongensis*	GD	China: Jiangxi: Mt Jinpen (41)	SYS a004456	ON391903	ON381892	√
102	*Nidirana guangdongensis*	GD	China: Jiangxi: Mt Jinpen (41)	SYS a004466	ON391904	ON381893	√
103	*Nidirana guangdongensis*	GD	China: Jiangxi: Mt Jiulian (42)	SYS a004060	ON391898	ON381887	√
104	*Nidirana guangdongensis*	GD	China: Jiangxi: Mt Jiulian (42)	SYS a004070	ON391899	ON381888	√
105	*Nidirana guangdongensis*	GD	China: Jiangxi: Mt Jiulian (42)	SYS a004071	MN946422	MN945178	√
106	*Nidirana guangdongensis*	GD	China: Jiangxi: Mt Jiulian (42)	SYS a004082	MN946423	MN945179	√
107	*Nidirana guangdongensis*	GD	China: Hunan: Mt Bamian (51)	SYS a004429	ON391902	ON381891	√
108	*Nidirana guangdongensis*	GD	China: Hunan: Mt Bamian (51)	SYS a006195	MN946420	MN945176	√
109	*Nidirana guangdongensis*	GD	China: Hunan: Mt Bamian (51)	SYS a006196	MN946421	MN945177	√
110	*Nidirana guangdongensis*	GD	China: Hunan: Mt Bamian (51)	SYS a006197	ON391918	ON381907	√

ID	Species	Population	Locality (Locality ID)	Voucher	16s rRNA	CO1	RAD-seq
111	*Nidirana guangdongensis*	GD	China: Hunan: Lake Dongjiang (52)	SYS a006519	ON391919	ON381908	
112	*Nidirana guangdongensis*	GD	China: Hunan: Lake Dongjiang (52)	SYS a006520	ON391920	ON381909	
113	*Nidirana guangdongensis*	GD	China: Hunan: Lake Dongjiang (52)	SYS a006522	ON391921	ON381910	
114	*Nidirana guangxiensis*	GX	China: Guangxi: Mt Daming (60)	SYS a008553	MZ677222	MZ678729	
115	*Nidirana guangxiensis*	GX	China: Guangxi: Mt Daming (60)	SYS a008554	MZ677223	MZ678730	
116	*Nidirana guangxiensis*	GX	China: Guangxi: Mt Daming (60)	SYS a008555	MZ677224	MZ678731	
117	*Nidirana guangxiensis*	GX	China: Guangxi: Mt Daming (60)	SYS a008556	MZ677225	MZ678732	
118	*Nidirana guangxiensis*	GX	China: Guangxi: Mt Daming (60)	SYS a008557	MZ677226	MZ678733	
119	*Nidirana hainanensis*	HN	China: Hainan: Mt Diaoluo (75)	SYS a003741	MF807821	MF807860	
120	*Nidirana hainanensis*	HN	China: Hainan: Mt Diaoluo (75)	SYS a007669	MN946451	MN945207	√
121	*Nidirana hainanensis*	HN	China: Hainan: Mt Diaoluo (75)	SYS a007670	MN946452	MN945208	√
122	*Nidirana hainanensis*	HN	China: Hainan: Mt Diaoluo (75)	SYS a007671	ON391810	ON381801	√
123	*Nidirana leishanensis* (*Nidirana* "*guibeiensis*")	LS	China: Hunan: Mt Shunhuang (55)	SYS a007256	ON391815	ON381806	√
124	*Nidirana leishanensis* (*Nidirana* "*guibeiensis*")	LS	China: Hunan: Mt Shunhuang (55)	SYS a007255	ON391816	ON381807	√
125	*Nidirana leishanensis* (*Nidirana* "*guibeiensis*")	LS	China: Hunan: Mt Shunhuang (55)	SYS a007257	ON391817	ON381808	√
126	*Nidirana leishanensis*	LS	China: Hunan: Suining (56)	SYS a007238	ON391811	ON381802	√
127	*Nidirana leishanensis*	LS	China: Hunan: Suining (56)	SYS a007239	ON391812	ON381803	√
128	*Nidirana leishanensis*	LS	China: Hunan: Suining (56)	SYS a007240	ON391813	ON381804	√
129	*Nidirana leishanensis*	LS	China: Hunan: Suining (56)	SYS a007249	ON391814	ON381805	√
130	*Nidirana leishanensis*	LS	China: Hunan: Tongdao (57)	CIB WB2020215	MZ707769	/	
131	*Nidirana leishanensis*	LS	China: Hunan: Tongdao (57)	CIB WB2020216	MZ707770	/	
132	*Nidirana leishanensis*	LS	China: Hunan: Tongdao (57)	CIB WB2020217	MZ707771	/	
133	*Nidirana leishanensis*	LS	China: Guizhou: Mt Leigong (66)	SYS a007908	MN946453	MN945209	√
134	*Nidirana leishanensis*	LS	China: Guizhou: Mt Fanjing (67)	SYS a007195	MN946454	MN945210	√
135	*Nidirana leishanensis*	LS	China: Guizhou: Mt Fanjing (67)	SYS a007196	MN946455	MN945211	√
136	*Nidirana leishanensis*	LS	China: Guangxi: Rongshui (65)	SYS a008558	MZ677227	MZ678734	
137	*Nidirana leishanensis*	LS	China: Guangxi: Rongshui (65)	SYS a008559	MZ677228	MZ678735	

ID	Species	Population	Locality (Locality ID)	Voucher	16s rRNA	CO1	RAD-seq
138	*Nidirana leishanensis*	LS	China: Guangxi: Rongshui (65)	SYS a008560	MZ677229	MZ678736	
139	*Nidirana leishanensis* (*Nidirana* "*guibeiensis*")	LS	China: Guangxi: Ziyuan (62)	NNU 00769	ON985177	ON968959	
140	*Nidirana leishanensis* (*Nidirana* "*guibeiensis*")	LS	China: Guangxi: Ziyuan (62)	NNU 00770	ON985178	ON968960	
141	*Nidirana leishanensis* (*Nidirana* "*guibeiensis*")	LS	China: Guangxi: Xing'an (63)	NNU 00810	ON985179	ON968961	
142	*Nidirana leishanensis* (*Nidirana* "*guibeiensis*")	LS	China: Guangxi: Mt Maoer (64)	NNU 00917	ON985180	ON968962	
143	*Nidirana leishanensis* (*Nidirana* "*guibeiensis*")	LS	China: Guangxi: Mt Maoer (64)	NNU 00918	ON985181	ON968963	
144	*Nidirana lini*	L	China: Yunnan: Lyuchun (85)	HNNU LC001	KF185066	/	
145	*Nidirana lini*	L	China: Yunnan: Jiangcheng (86)	SYS a003967	MF807818	MF807857	√
146	*Nidirana lini*	L	China: Yunnan: Jiangcheng (86)	SYS a003968	MF807819	MF807858	√
147	*Nidirana lini*	L	China: Yunnan: Jiangcheng (86)	SYS a003969	MF807820	MF807859	
148	*Nidirana lini*	L	China: Yunnan: Jiangcheng (86)	SYS a003970	ON391818	ON381809	√
149	*Nidirana lini*	L	Laos: Xieng Khouang (88)	FMNH 256531	KR264073	/	
150	*Nidirana lini*	L	Laos: Xieng Khouang (88)	FMNH 256532	KR264074	/	
151	*Nidirana mangveni*	MV	China: Zhejiang: Suichang (17)	SYS a008037	ON391843	ON381834	√
152	*Nidirana mangveni*	MV	China: Zhejiang: Suichang (17)	SYS a008038	ON391844	ON381835	√
153	*Nidirana mangveni*	MV	China: Zhejiang: Suichang (17)	SYS a008040	ON391845	ON381836	√
154	*Nidirana mangveni*	MV	China: Zhejiang: Suichang (17)	SYS a008043	ON391846	ON381837	√
155	*Nidirana mangveni*	MV	China: Zhejiang: Qujiang (18)	SYS a008058	ON391850	ON381841	
156	*Nidirana mangveni*	MV	China: Zhejiang: Qujiang (18)	SYS a008059	ON391851	ON381842	
157	*Nidirana mangveni*	MV	China: Zhejiang: Qujiang (18)	SYS a008060	ON391852	ON381843	
158	*Nidirana mangveni*	MV	China: Zhejiang: Longyou (19)	SYS a008050	ON391847	ON381838	
159	*Nidirana mangveni*	MV	China: Zhejiang: Longyou (19)	SYS a008051	ON391848	ON381839	
160	*Nidirana mangveni*	MV	China: Zhejiang: Longyou (19)	SYS a008052	ON391849	ON381840	
161	*Nidirana mangveni*	MV	China: Zhejiang: Mt Jinhua (20)	SYS a008007	ON391837	ON381828	
162	*Nidirana mangveni*	MV	China: Zhejiang: Mt Jinhua (20)	SYS a008008	ON391838	ON381829	
163	*Nidirana mangveni*	MV	China: Zhejiang: Mt Jinhua (20)	SYS a008009	ON391839	ON381830	

ID	Species	Population	Locality (Locality ID)	Voucher	16s rRNA	CO1	RAD-seq
164	*Nidirana mangveni*	MV	China: Zhejiang: Yongkang (21)	SYS a008032	ON391840	ON381831	√
165	*Nidirana mangveni*	MV	China: Zhejiang: Yongkang (21)	SYS a008033	ON391841	ON381832	√
166	*Nidirana mangveni*	MV	China: Zhejiang: Yongkang (21)	SYS a008034	ON391842	ON381833	√
167	*Nidirana mangveni*	MV	China: Zhejiang: Mt Dapan (22)	SYS a006310	MN946424	MN945180	√
168	*Nidirana mangveni*	MV	China: Zhejiang: Mt Dapan (22)	SYS a006311	MN946425	MN945181	√
169	*Nidirana mangveni*	MV	China: Zhejiang: Mt Dapan (22)	SYS a006312	MN946426	MN945182	√
170	*Nidirana mangveni*	MV	China: Zhejiang: Jiande (23)	SYS a007998	ON391834	ON381825	
171	*Nidirana mangveni*	MV	China: Zhejiang: Jiande (23)	SYS a007999	ON391835	ON381826	
172	*Nidirana mangveni*	MV	China: Zhejiang: Jiande (23)	SYS a008000	ON391836	ON381827	
173	*Nidirana mangveni*	MV	China: Zhejiang: Mt Longmen (24)	SYS a006413	MN946428	MN945184	√
174	*Nidirana mangveni*	MV	China: Zhejiang: Mt Longmen (24)	SYS a006414	MN946429	MN945185	√
175	*Nidirana mangveni*	MV	China: Zhejiang: Mt Longmen (24)	SYS a006415	MN946430	MN945186	√
176	*Nidirana mangveni*	MV	China: Zhejiang: Hangzhou (25)	SYS a007992	ON391831	ON381822	√
177	*Nidirana mangveni*	MV	China: Zhejiang: Hangzhou (25)	SYS a007993	ON391832	ON381823	√
178	*Nidirana mangveni*	MV	China: Zhejiang: Hangzhou (25)	SYS a007994	ON391833	ON381824	√
179	*Nidirana mangveni*	MV	China: Anhui: Mt Qingliangfeng (26)	SYS a007955	ON391828	ON381819	√
180	*Nidirana mangveni*	MV	China: Anhui: Mt Qingliangfeng (26)	SYS a007956	ON391829	ON381820	√
181	*Nidirana mangveni*	MV	China: Anhui: Mt Qingliangfeng (26)	SYS a007957	ON391830	ON381821	√
182	*Nidirana mangveni*	MV	China: Anhui: Xiuning (27)	SYS a007933	ON391825	ON381816	√
183	*Nidirana mangveni*	MV	China: Anhui: Xiuning (27)	SYS a007934	ON391826	ON381817	√
184	*Nidirana mangveni*	MV	China: Anhui: Xiuning (27)	SYS a007935	ON391827	ON381818	√
185	*Nidirana mangveni*	MV	China: Jiangxi: Mt Taohongling (28)	SYS a007888	ON391823	ON381814	√
186	*Nidirana mangveni*	MV	China: Jiangxi: Mt Taohongling (28)	SYS a007889	ON391824	ON381815	√
187	*Nidirana mangveni*	MV	China: Jiangxi: Mt Sanqing (29)	SYS a008070	ON391853	ON381844	√
188	*Nidirana mangveni*	MV	China: Jiangxi: Mt Sanqing (29)	SYS a008071	ON391854	ON381845	√
189	*Nidirana mangveni*	MV	China: Jiangxi: Mt Sanqing (29)	SYS a008072	ON391855	ON381846	√
190	*Nidirana mangveni*	MV	China: Jiangxi: Yanshan (30)	SYS a007340	ON391820	ON381811	√
191	*Nidirana mangveni*	MV	China: Jiangxi: Yanshan (30)	SYS a007341	ON391821	ON381812	√
192	*Nidirana mangveni*	MV	China: Jiangxi: Yanshan (30)	SYS a007342	ON391822	ON381813	√

ID	Species	Population	Locality (Locality ID)	Voucher	16s rRNA	CO1	RAD-seq
193	*Nidirana mangveni*	MV	China: Jiangxi: Yanshan (30)	SYS a008089	ON391856	ON381847	√
194	*Nidirana mangveni*	MV	China: Jiangxi: Yanshan (30)	SYS a008090	ON391857	ON381848	√
195	*Nidirana mangveni*	MV	China: Jiangxi: Yanshan (30)	SYS a008091	ON391858	ON381849	√
196	*Nidirana mangveni*	MV	China: Jiangxi: Yanshan (30)	SYS a008100	ON391859	ON381850	√
197	*Nidirana mangveni*	MV	China: Jiangxi: Yanshan (30)	SYS a008102	ON391860	ON381851	√
198	*Nidirana mangveni*	MV	China: Jiangxi: Yanshan (30)	SYS a008104	ON391861	ON381852	√
199	*Nidirana mangveni*	MV	China: Jiangxi: Yanshan (30)	SYS a008105	ON391862	ON381853	√
200	*Nidirana mangveni*	MV	China: Jiangxi: Dongxiang (31)	SYS a008111	ON391863	ON381854	√
201	*Nidirana mangveni*	MV	China: Jiangxi: Dongxiang (31)	SYS a008112	ON391864	ON381855	√
202	*Nidirana mangveni*	MV	China: Jiangxi: Dongxiang (31)	SYS a008113	ON391865	ON381856	√
203	*Nidirana nankunensis*	N	China: Guangdong: Mt Nankun (45)	SYS a004905	ON391866	ON381857	√
204	*Nidirana nankunensis*	N	China: Guangdong: Mt Nankun (45)	SYS a004906	MF807835	MF807874	√
205	*Nidirana nankunensis*	N	China: Guangdong: Mt Nankun (45)	SYS a004907	MF807836	MF807875	√
206	*Nidirana nankunensis*	N	China: Guangdong: Mt Nankun (45)	SYS a004914	MF807837	MF807876	
207	*Nidirana nankunensis*	N	China: Guangdong: Mt Nankun (45)	SYS a005717	MF807838	MF807877	
208	*Nidirana occidentalis*	OC	China: Yunnan: Mt Gaoligong (83)	SYS a003775	MF807816	MF807855	√
209	*Nidirana occidentalis*	OC	China: Yunnan: Mt Gaoligong (83)	SYS a003776	MF807817	MF807856	
210	*Nidirana occidentalis*	OC	China: Yunnan: Shuangjiang (84)	SYS a007829	MT935679	MT932859	√
211	*Nidirana occidentalis*	OC	China: Yunnan: Shuangjiang (84)	SYS a007830	MT935680	MT932860	√
212	*Nidirana occidentalis*	OC	China: Yunnan: Shuangjiang (84)	SYS a007831	MT935681	MT932861	√
213	*Nidirana occidentalis*	OC	China: Yunnan: Shuangjiang (84)	SYS a007832	MT935682	MT932862	
214	*Nidirana okinavana*	OK	Japan: Okinawa: Iriomote (3)	SYS a007606	ON391819	ON381810	√
215	*Nidirana okinavana*	OK	Japan: Okinawa: Iriomote (3)	NA	NC022872	NC022872	
216	*Nidirana pleuraden*	Pe	China: Guizhou: Lake Caohai (69)	SYS a006598	ON391804	ON381795	
217	*Nidirana pleuraden*	Pe	China: Guizhou: Lake Caohai (69)	SYS a006599	ON391805	ON381796	
218	*Nidirana pleuraden*	Pe	China: Guizhou: Lake Caohai (69)	SYS a006600	ON391806	ON381797	
219	*Nidirana pleuraden*	Pe	China: Guizhou: Shuicheng (70)	SYS a007694	ON391807	ON381798	√
220	*Nidirana pleuraden*	Pe	China: Guizhou: Shuicheng (70)	SYS a007695	ON391808	ON381799	√
221	*Nidirana pleuraden*	Pe	China: Guizhou: Shuicheng (70)	SYS a007696	ON391809	ON381800	√

ID	Species	Population	Locality (Locality ID)	Voucher	16s rRNA	CO1	RAD-seq
222	*Nidirana pleuraden*	Pe	China: Guizhou: Anlong (71)	SYS a008305	ON391801	ON381792	√
223	*Nidirana pleuraden*	Pe	China: Guizhou: Anlong (71)	SYS a008306	ON391802	ON381793	√
224	*Nidirana pleuraden*	Pe	China: Guizhou: Anlong (71)	SYS a008307	ON391803	ON381794	√
225	*Nidirana pleuraden*	Pw	China: Yunnan: Kunming (77)	SYS a007858	MT935683	MT932858	√
226	*Nidirana pleuraden*	Pw	China: Yunnan: Wenshan (79)	SYS a007717	MT935671	MT932850	√
227	*Nidirana pleuraden*	Pw	China: Yunnan: Wenshan (79)	SYS a007718	MT935672	MT932851	√
228	*Nidirana pleuraden*	Pw	China: Yunnan: Wenshan (79)	SYS a007719	MT935673	MT932852	√
229	*Nidirana pleuraden*	Pw	China: Yunnan: Shiping (80)	SYS a007786	ON391798	ON381789	√
230	*Nidirana pleuraden*	Pw	China: Yunnan: Shiping (80)	SYS a007787	ON391799	ON381790	√
231	*Nidirana pleuraden*	Pw	China: Yunnan: Shiping (80)	SYS a007788	ON391800	ON381791	√
232	*Nidirana pleuraden*	Pw	China: Yunnan: Xinping (81)	SYS a007767	ON391795	ON381786	√
233	*Nidirana pleuraden*	Pw	China: Yunnan: Xinping (81)	SYS a007768	ON391796	ON381787	√
234	*Nidirana pleuraden*	Pw	China: Yunnan: Xinping (81)	SYS a007769	ON391797	ON381788	√
235	*Nidirana pleuraden*	Pw	China: Yunnan: Lijiang (82)	CIB XM2958	KF771283	KF771326	
236	*Nidirana shiwandashanensis*	SW	China: Guangxi: Mt Shiwandashan (61)	NNU 00605	OK383411	OK377265	
237	*Nidirana shiwandashanensis*	SW	China: Guangxi: Mt Shiwandashan (61)	NNU 00606	OK383412	OK377266	
238	*Nidirana shiwandashanensis*	SW	China: Guangxi: Mt Shiwandashan (61)	NNU 00600	OK383413	OK377267	
239	*Nidirana xiangica*	X	China: Jiangxi: Mt Wugong (37)	SYS a002590	MN946441	MN945197	√
240	*Nidirana xiangica*	X	China: Hunan: Mt Dawei (53)	SYS a006491	MN946433	MN945189	√
241	*Nidirana xiangica*	X	China: Hunan: Mt Dawei (53)	SYS a006492	MN946434	MN945190	√
242	*Nidirana xiangica*	X	China: Hunan: Mt Dawei (53)	SYS a006493	MN946435	MN945191	√
243	*Nidirana xiangica*	X	China: Hunan: Mt Yangming (54)	SYS a007269	MN946436	MN945192	√
244	*Nidirana xiangica*	X	China: Hunan: Mt Yangming (54)	SYS a007270	MN946437	MN945193	√
245	*Nidirana xiangica*	X	China: Hunan: Mt Yangming (54)	SYS a007271	MN946438	MN945194	√
246	*Nidirana xiangica*	X	China: Guangxi: Mt Dupangling (58)	SYS a006563	ON391886	ON381875	√
247	*Nidirana xiangica*	X	China: Guangxi: Mt Dupangling (58)	SYS a006564	ON391887	ON381876	√
248	*Nidirana xiangica*	X	China: Guangxi: Mt Dupangling (58)	SYS a006565	ON391888	ON381877	√
249	*Nidirana xiangica*	X	China: Guangxi: Mt Dupangling (58)	SYS a006566	ON391889	ON381878	√
250	*Nidirana yaoica*	Y	China: Guangxi: Mt Dayao (59)	SYS a007011	MK882272	MK895037	√

ID	Species	Population	Locality (Locality ID)	Voucher	16s rRNA	CO1	RAD-seq
251	*Nidirana yaoica*	Y	China: Guangxi: Mt Dayao (59)	SYS a007012	MK882273	MK895038	√
252	*Nidirana yaoica*	Y	China: Guangxi: Mt Dayao (59)	SYS a007013	MK882274	MK895039	√
253	*Nidirana yaoica*	Y	China: Guangxi: Mt Dayao (59)	SYS a007014	MK882275	MK895040	√
254	*Nidirana yaoica*	Y	China: Guangxi: Mt Dayao (59)	SYS a007020	MK882276	MK895041	
255	*Nidirana yeae*	YE	China: Guizhou: Tongzi (68)	SYS a008266	ON391890	ON381879	√
256	*Nidirana yeae*	YE	China: Guizhou: Tongzi (68)	SYS a008267	ON391891	ON381880	√
257	*Nidirana yeae*	YE	China: Guizhou: Tongzi (68)	SYS a008268	ON391892	ON381881	√
258	*Babina holsti*	B	Japan: Okinawa: Okinawa (2)	NA	NC022870	NC022870	
259	*Babina holsti*	B	Japan: Okinawa: Okinawa (2)	SYS a007608	/	/	√
260	*Babina subaspera*	B	Japan: Kagoshima: Amami (1)	NA	NC022871	NC022871	
261	*Babina subaspera*	B	Japan: Kagoshima: Amami (1)	SYS a007607	/	/	√
262	*Odorrana graminea*	O	China: Hainan: Mt Wuzhi (76)	SYS a005271	ON391778	ON381769	√
263	*Odorrana margaratae*	O	China: Sichuan: Mt Emei (74)	SYS a005303	ON391779	ON381770	√

Supplementary Table S2 Adjacent geographical areas allowed for ancestral ranges estimating.

ID	Area 1	Geographical barrier	Area 2
1	A	Langcang River	B
2	A	Red River and Ailao Mountains	C
3	A	Red River and Ailao Mountains	F
4	A	Beibu Gulf	J
5	B	Langcang River	C
6	C	Daliang Mountains – Dalou Mountains	D
7	C	Wuling Mountains	E
8	C	Miaoling Mountains	F
9	D	Wushan Mountains	E
10	E	Nanling Mountains	F
11	E	Wuyi Mountains and Xinjiang River	G
12	E	Nanling Mountains	H
13	F	Yunkai Mountains	H
14	F	Beibu Gulf	J
15	G	Minjiang River	H
16	G	Taiwan Strait	I
17	H	Taiwan Strait	I
18	H	Qiongzhou Strait	J
19	I	South China Sea	J

Details for the ten areas:
(A) tropical hills in southern Yunnan and northern Indochina Peninsula;
(B) longitudinal valleys in western Yunnan;
(C) Yunnan-Guizhou Plateau, encompassing eastern Yunnan and most of Guizhou;
(D) Sichuan Basin;
(E) Luoxiao Mountain Range and nonboring river basin;
(F) Guangxi Basin;
(G) Zhejiang-Fujian hills
(H) costal hills of Guangdong and southern Fujian;
(I) Taiwan Island and southern Ryukyu Islands.
(J) Hainan Island;

Supplementary Table S3 The four scenarios modeled for three pairs of *Nidirana* populations using fastsimcoal2. The best scenario model for each populations pair is highlighted in bold.

Model				
	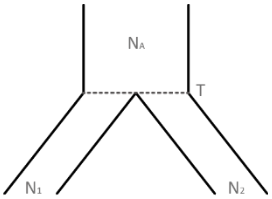	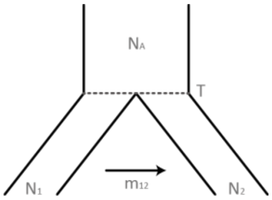	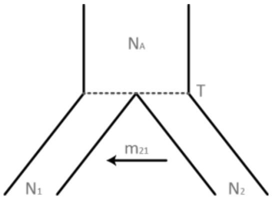	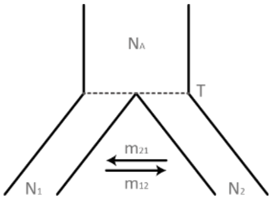
	mig0	mig12	mig21	mig2
		**An (pop1) vs. MV (pop2)**		
Parameters	4	5	5	**6**
MaxEstLhood	-85798.382	-85768.953	-85788.731	**-85719.982**
MaxObsLhood	-76846.849	-76846.849	-76846.849	**-76846.849**
AIC	171604.764	171547.906	171587.462	**171451.964**
delta AIC	-152.8	-95.942	-135.498	**0**
NA	78565476	78795987	78641142	**79196307**
N1	2085552	1770247	1555996	**1165424**
N2	7415514	7346615	6249100	**4392061**
T	95641	84537	73350	**55060**
M12	0	1.67e-08	0	**2.22e-08**
M21	0	0	3.00e-08	**1.21e-07**
		**An (pop1) vs. Ac (pop2)**		
Parameters	4	5	**5**	6
MaxEstLhood	-77686.363	-77687.133	**-77676.994**	-77782.669
MaxObsLhood	-66784.034	-66784.034	**-66784.034**	-66784.034
AIC	155380.726	155384.266	**155363.988**	155577.338
delta AIC	-16.738	-20.278	**0**	-213.35
NA	78605893	78590954	**78664861**	79109957
N1	5909266	6582263	**6184312**	3111643
N2	1845078	1893909	**1921762**	14161
T	217189	214531	**219319**	4444
M12	0	3.46e-09	**0**	1.93e-04
M21	0	0	**6.60e-09**	7.74e-07
		**At (pop1) vs. MV (pop2)**		
Parameters	**4**	5	5	6
MaxEstLhood	**-62570.59**	-62703.776	-62598.462	-62671.087
MaxObsLhood	**-54440.859**	-54440.859	-54440.859	-54440.859
AIC	**125149.18**	125417.552	125206.924	125354.174
delta AIC	**0**	-268.372	-57.744	-204.994
NA	**78546459**	78450546	78684063	78905015
N1	**185215**	93629	596	34328
N2	**15243846**	8632663	1250152	3328071
T	**275589**	132711	1090	48774
M12	**0**	1.12E-07	0	3.09E-07
M21	**0**	0	1.15E-04	9.53E-08

Supplementary Table S4 Correlation of nidification behavior with the 19 bioclimatic variables. Rank by *P* value from lowest to highest. * *P* values < 0.05, ** *P* values < 0.01.

Rank	Bioclimatic variables	P value
**1**	**Bio7**	**0.004****
**2**	**Bio4**	**0.004****
**3**	**Bio15**	**0.007****
4	Bio18	0.012*
5	Bio19	0.012*
6	Bio6	0.012*
7	Bio2	0.017*
8	Bio11	0.017*
9	Bio3	0.017*
10	Bio8	0.017*
11	Bio5	0.034*
12	Bio17	0.034*
13	Bio10	0.126
14	Bio9	0.186
15	Bio1	0.196
16	Bio16	0.229
17	Bio14	0.234
18	Bio13	0.378
19	Bio12	0.881

Bio1 = Annual Mean Temperature
Bio2 = Mean Diurnal Range (Mean of monthly (max temp - min temp))
Bio3 = Isothermality (Bio2/Bio7) (×100)
Bio4 = Temperature Seasonality (standard deviation ×100)
Bio5 = Max Temperature of Warmest Month
Bio6 = Min Temperature of Coldest Month
Bio7 = Temperature Annual Range (Bio5-Bio6)
Bio8 = Mean Temperature of Wettest Quarter
Bio9 = Mean Temperature of Driest Quarter
Bio10 = Mean Temperature of Warmest Quarter
Bio11 = Mean Temperature of Coldest Quarter
Bio12 = Annual Precipitation
Bio13 = Precipitation of Wettest Month
Bio14 = Precipitation of Driest Month
Bio15 = Precipitation Seasonality (Coefficient of Variation)
Bio16 = Precipitation of Wettest Quarter
Bio17 = Precipitation of Driest Quarter
Bio18 = Precipitation of Warmest Quarter
Bio19 = Precipitation of Coldest Quarter
