# Supplementary material for: Contrasting nidification behaviors facilitate diversification and colonization of the Music frogs under a changing paleoclimate
Source: Commun Biol. 2024 May 25;7:638. doi: 10.1038/s42003-024-06347-7 (PMC11127999; doi:10.1038/s42003-024-06347-7)
Supplement: Supplementary file 3 — Reporting Summary [file 42003_2024_6347_MOESM3_ESM.pdf]

nature portfolio

Corresponding author(s): Yang Liu & Ying-Yong Wang

Last updated by author(s): Apr 28, 2024

Reporting Summary

Nature Portfolio wishes to improve the reproducibility of the work that we publish. This form provides structure for consistency and transparency in reporting. For further information on Nature Portfolio policies, see our Editorial Policies and the Editorial Policy Checklist.

Statistics

For all statistical analyses, confirm that the following items are present in the figure legend, table legend, main text, or Methods section.

n/a	Confirmed	
☐	☒	The exact sample size (*n*) for each experimental group/condition, given as a discrete number and unit of measurement
☒	☐	A statement on whether measurements were taken from distinct samples or whether the same sample was measured repeatedly
☒	☐	The statistical test(s) used AND whether they are one- or two-sided<br>*Only common tests should be described solely by name; describe more complex techniques in the Methods section.*
☒	☐	A description of all covariates tested
☒	☐	A description of any assumptions or corrections, such as tests of normality and adjustment for multiple comparisons
☒	☐	A full description of the statistical parameters including central tendency (e.g. means) or other basic estimates (e.g. regression coefficient) AND variation (e.g. standard deviation) or associated estimates of uncertainty (e.g. confidence intervals)
☐	☒	For null hypothesis testing, the test statistic (e.g. *F*, *t*, *r*) with confidence intervals, effect sizes, degrees of freedom and *P* value noted<br>*Give P values as exact values whenever suitable.*
☒	☐	For Bayesian analysis, information on the choice of priors and Markov chain Monte Carlo settings
☒	☐	For hierarchical and complex designs, identification of the appropriate level for tests and full reporting of outcomes
☒	☐	Estimates of effect sizes (e.g. Cohen's *d*, Pearson's *r*), indicating how they were calculated

Our web collection on statistics for biologists contains articles on many of the points above.

Software and code

Policy information about availability of computer code

Data collection	WorldClim v2.1
Data analysis	Stacks v2.53, VCFtools v0.1.16, MEGA 6, jmodeltest v2.1.7, RAxML v8.0, MrBayes 3.2.4, Tracer v1.7, vcf2phylip (https://github.com/edgardomortiz/vcf2phylip), RAxML-NG, PLINK 1.9, PAML v4.8, RevBayes, BEAST v2.5, Admixture 1.3, Stairway Plot 2, easySFS (https://github.com/isaacovercast/easySFS), fastsimcoal v2.7, and R-4.2.1

For manuscripts utilizing custom algorithms or software that are central to the research but not yet described in published literature, software must be made available to editors and reviewers. We strongly encourage code deposition in a community repository (e.g. GitHub). See the Nature Portfolio guidelines for submitting code & software for further information.

Data

Policy information about availability of data

All manuscripts must include a data availability statement. This statement should provide the following information, where applicable:

- Accession codes, unique identifiers, or web links for publicly available datasets
- A description of any restrictions on data availability
- For clinical datasets or third party data, please ensure that the statement adheres to our policy

All mitochondrial DNA Sanger sequencing data were uploaded to NCBI Nucleotide Database and the accession numbers are provided in Supplementary Table S1. All

raw sequencing data generated by ddRAD-seq were uploaded to NCBI Sequence Read Archive under the BioProject accession number PRJNA1091219. Alignment for phylogeny and supplementary tables and figures are available on at https://doi.org/10.5061/dryad.wstqjq2rs.

Human research participants

Policy information about studies involving human research participants and Sex and Gender in Research.

Reporting on sex and gender	Not involving human participants, their data, or biological material.
Population characteristics	Not involving human participants, their data, or biological material.
Recruitment	Not involving human participants, their data, or biological material.
Ethics oversight	Not involving human participants, their data, or biological material.

Note that full information on the approval of the study protocol must also be provided in the manuscript.

Field-specific reporting

Please select the one below that is the best fit for your research. If you are not sure, read the appropriate sections before making your selection.

☐ Life sciences ☐ Behavioural & social sciences ☒ Ecological, evolutionary & environmental sciences

For a reference copy of the document with all sections, see nature.com/documents/nr-reporting-summary-flat.pdf

Ecological, evolutionary & environmental sciences study design

All studies must disclose on these points even when the disclosure is negative.

Study description	To understand the evolutionary history of the Music frogs and their unique mud nest construction behavior, we carried out a series of phylogenomic and population genomic analyses based on an unprecedented sampling of all described species of the genus. We hypothesized that the presence and loss of nidification behavior are associated with ancient climate changes and that this behavior further acts as a key innovation that contributed to the diversification and colonization of these frogs. Firstly, we employed genome-wide single-nucleotide polymorphisms (SNPs) to investigate their time-calibrated phylogeny and reconstruct their ancestral distributions. Secondly, to uncover the speciation of related species, we performed analyses on population genetics and demography for the congener subset with or without nidification behavior. Moreover, we assessed the correlation and dependence of the nidification behavior with several related bioclimatic factors.
Research sample	Field surveys were conducted throughout the distribution range of the genus Nidirana according to previous reports on their distributions. This sampling has involved all known species of the genus and their geogarphical populations.
Sampling strategy	Field surveys were conducted throughout the distribution range of the genus Nidirana according to previous reports on their distributions. This sampling has involved all known species of the genus and their geogarphical populations.
Data collection	Experiment for DNA extraction was conducted by ZT Lyu, PCR for mitochondrial sequences was conducted by ZT Lyu, mitochondiral sequencing and ddRAD-seq were conducted by Shenzhen RealOmics (Biotech) Co., Ltd. Detailed data collection procedure has been provided in the manuscript.
Timing and spatial scale	Field surveys were conducted during the breeding seasons of the Nidirana frogs from 2014 to 2021, throughout the distribution range of the genus Nidirana (Fig. 1c; Supplementary Table S1).
Data exclusions	No data were excluded from the analyses.
Reproducibility	Underlying data and supplementary tables and figures are available on at https://doi.org/10.5061/dryad.wstqjq2rs, to verify the reproducibility of the analyses.
Randomization	All samples were allocated into groups based on the morphological identifications.
Blinding	Samples of Babina and Odorrana species used as out-groups according to their phylogenetic relationships.

Did the study involve field work? ☒ Yes ☐ No

Field work, collection and transport

Field conditions	The field works were conducted at night and the temperature was at 20-30 °C.
Location	All location data of sampling is provieded in the Supplementary Fig. S1.
Access & import/export	The collection of samples was performed within a long-term investigation project on the specimen platform of China, teaching specimen sub-platform (http://mnh.scu.edu.cn/) to YY Wang, and all the procedures related animals were performed in accordance with the ethical guidelines and approval of the of Institutional Animal Care and Use Committee of Sun Yat-sen University (2005DKA21403-JK).
Disturbance	Human activity was the major disturbance and it was minimized by strictly following the criterion for field sampling.

Reporting for specific materials, systems and methods

We require information from authors about some types of materials, experimental systems and methods used in many studies. Here, indicate whether each material, system or method listed is relevant to your study. If you are not sure if a list item applies to your research, read the appropriate section before selecting a response.

Materials & experimental systems

n/a	Involved in the study
☒	☐ Antibodies
☒	☐ Eukaryotic cell lines
☒	☐ Palaeontology and archaeology
☐	☒ Animals and other organisms
☒	☐ Clinical data
☒	☐ Dual use research of concern

Methods

n/a	Involved in the study
☒	☐ ChIP-seq
☒	☐ Flow cytometry
☒	☐ MRI-based neuroimaging

Animals and other research organisms

Policy information about studies involving animals; ARRIVE guidelines recommended for reporting animal research, and Sex and Gender in Research

Laboratory animals	The study did not involve laboratory animals.
Wild animals	All individuals were captured in the field by hand. All specimens were fixed in 10% buffered formalin and later transferred to 70% ethanol; muscle or liver samples were obtained from euthanized specimens and then preserved in 95% ethanol and stored at −40 °C in the Museum of Biology, Sun Yat-sen University.
Reporting on sex	This study is not involving on sex control.
Field-collected samples	The study did not involve samples collected from the field.
Ethics oversight	All the procedures related animals were performed in accordance with the ethical guidelines and approval of the of Institutional Animal Care and Use Committee of Sun Yat-sen University (2005DKA21403-JK).

Note that full information on the approval of the study protocol must also be provided in the manuscript.
